# Integrated analysis reveals the alterations that LMNA interacts with euchromatin in LMNA mutation-associated dilated cardiomyopathy

**DOI:** 10.1186/s13148-020-00996-1

**Published:** 2021-01-06

**Authors:** Xiaolin Zhang, Xiuli Shao, Ruijia Zhang, Rongli Zhu, Rui Feng

**Affiliations:** Department of Pharmaceutical Toxicology, School of Pharmacy, China Medical University, No.77 Puhe Road, Shenyang North New Area, Shenyang, 110122 China

**Keywords:** LMNA, Mutation, Dilated cardiomyopathy, WNT signaling, TGFβ-BMP signaling

## Abstract

**Background:**

Dilated cardiomyopathy (DCM) is a serious cardiac heterogeneous pathological disease, which may be caused by mutations in the LMNA gene. Lamins interact with not only lamina-associated domains (LADs) but also euchromatin by alone or associates with the lamina-associated polypeptide 2 alpha (LAP2α). Numerous studies have documented that LMNA regulates gene expression by interacting with LADs in heterochromatin. However, the role of LMNA in regulating euchromatin in DCM is poorly understood. Here, we determine the differential binding genes on euchromatin in DCM induced by LMNA mutation by performing an integrated analysis of bioinformatics and explore the possible molecular pathogenesis mechanism.

**Results:**

Six hundred twenty-three and 4484 differential binding genes were identified by ChIP-seq technology. The ChIP-seq analysis results and matched RNA-Seq transcriptome data were integrated to further validate the differential binding genes of ChIP-seq. Five and 60 candidate genes involved in a series of downstream analysis were identified. Finally, 4 key genes (CREBBP, PPP2R2B, BMP4, and BMP7) were harvested, and these genes may regulate LMNA mutation-induced DCM through WNT/β-catenin or TGFβ-BMP pathways.

**Conclusions:**

We identified four key genes that may serve as potential biomarkers and novel therapeutic targets. Our study also illuminates the possible molecular pathogenesis mechanism that the abnormal binding between LMNA or LAP2α-lamin A/C complexes and euchromatin DNA in LMNA mutations, which may cause DCM through the changes of CREBBP, PPP2R2B, BMP4, BMP7 expressions, and the dysregulation of WNT/β-catenin or TGFβ-BMP pathways, providing valuable insights to improve the occurrence and development of DCM.

**Graphic abstract:**

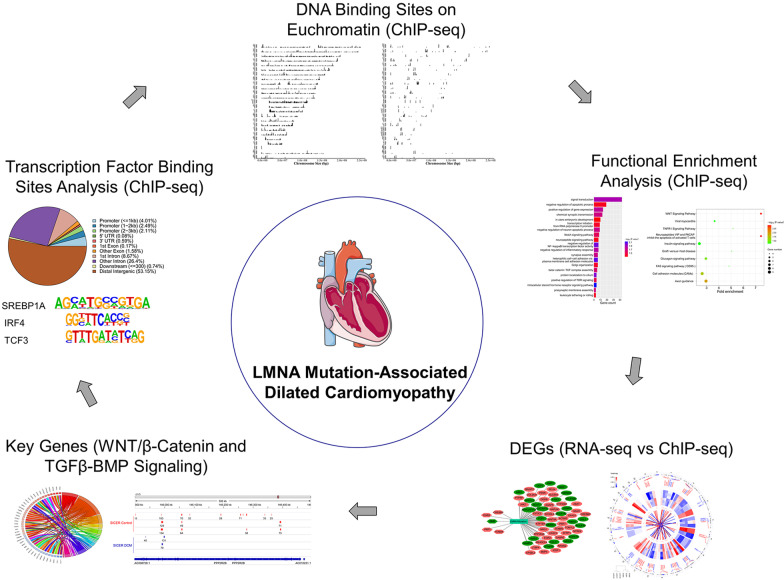

## Introduction

Lamins, evolutionarily conserved nuclear envelope proteins, are important for maintaining normal cell functions, including DNA replication, gene expression, chromatin organization, mitosis regulation, nuclear stability, and signal transduction [1]. Lamins, existing in the form of dimers, include three types type A, type B, and type C. In the lamin family, lamins A and C are encoded by the LMNA gene originated from the same transcription by alternative splicing [2]. Mutations of LMNA gene can cause a wide array of heart diseases, such as atrial arrhythmia, atrioventricular block, sinus bradycardia, and dilated cardiomyopathy (DCM). DCM is a severe cardiac heterogeneous pathology characterized by ventricular dilatation and systolic impairment, which is accompanied by thromboembolism, arrhythmia, and even sudden death [3]. DCM is also a leading cause of heart failure [4]. LMNA mutations can cause autosomal dominant inheritable cardiomyopathy, accounting for 10% of DCM. In comparison with other etiologies of DCM, LMNA mutation-associated DCM not only has high incidences and faster progress, but also is more malignant even when the ventricular impairment is mild [1]. At present, there are certain insights into the pathogenesis of LMNA mutation-associated DCM. However, due to the complexity of LMNA mutations, patients with the same mutation have different clinical characteristics. The phenotype-genotype correlation also increases the difficulty of LMNA mutation-associated DCM understanding. Therefore, our knowledge about the DCM mechanisms caused by LMNA mutation is vague and incomplete.

To date, multiple mutation sites of LMNA (lamin A/C) related to DCM have been reported, such as LMNA-D300N, LMNA-H222P, and LMNA-N195K [5–7]. Also, increasing researches support that LMNA mutations cause DCM through dysregulation of various molecular pathways, such as mitogen-activated protein kinases (MAPK) pathway, AKT/mTOR pathway, and WNT pathway [8, 9]. Previous evidences had shown that MAPK pathway and mammalian target of rapamycin complex 1 (mTORC1) pathway have abnormal activation in LMNA mutation induced-DCM [10–13].

LMNA, the component of nuclear inner membrane protein, is located mostly at the nuclear periphery [14]. Cheedipudi SM et al. reported that Lamin A/C interacted with heterochromatic lamina-associated domains (LADs) to regulate gene expression in DCM [9]. However, according to recent reports, the combination of LMNA and euchromatin attracts high attention, and the role of LMNA binding to euchromatin in LMNA mutation-associated DCM is still unclear. Although LMNA is mainly distributed around the nuclear periphery, it is less tightly linked to the inner nuclear membrane (INM). They are also found in a more mobile and dynamic throughout the nucleoplasm pool. LMNA can bind euchromatin directly or by the complex with lamina-associated polypeptide (LAP2α, a protein from the Lamina-associated polypeptide 2 family), which plays an important biological function [15]. LAP2α is a non-membrane protein associated with the nucleoskeleton and is uniformly distributed throughout the nucleoplasm [16]. It may help to dynamically associate with chromosomes by interacting with LMNA via their unique C-terminal tails [17]. The binding disorders of nucleoplasmic LAP2α-lamin A/C complexes cause late onset striated muscle diseases or erythroid and epidermal progenitor hyperproliferation, and the disorders also impair heart function and stress response [18, 19]. However, the regulation and specific functions of this dynamic, nucleoplasmic pool of lamin A/C are still poorly understood.

For the role of LMNA in regulating euchromatin in DCM, we speculate that it may result from the abnormal binding between LMNA alone or LAP2α-lamin A/C complexes and euchromatin in myocardial cells. To test this hypothesis, we tried to elucidate the potential biological processes and signaling pathways associated with LMNA mutation-associated DCM through deeply analyzing the chromatin immunoprecipitation sequencing (ChIP-seq) and RNA sequencing (RNA-seq) datasets, which were obtained from the Gene Expression Omnibus (GEO) database. Gene Ontology (GO) and pathway analysis were further conducted to assess the potential functions of the differential binding genes. The dysregulated hub genes and associated signaling pathways were also discovered in this study.

## Results

### The decreased DNA binding sites of euchromatin in LMNA mutation-associated DCM

**Fig. 1:**
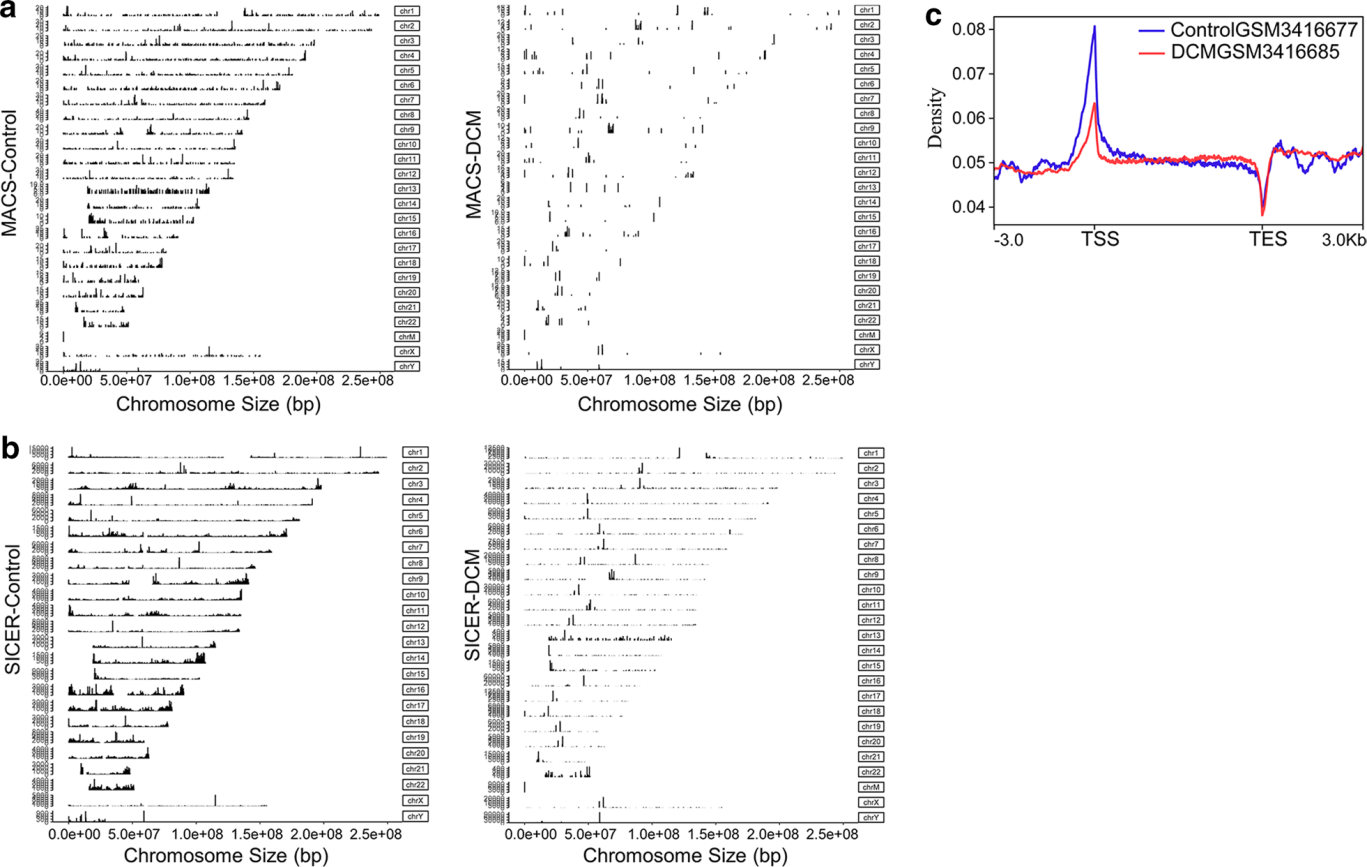
Visualization of the chromosomal binding patterns in control and DCM groups and genomic distribution. **a** Peak patterns and locations identified by MACS. **b** Peak patterns and locations identified by SICER. **c** Density plots of genomic signal distribution in gene body

We used principal component analysis (PCA) to compare the correlations among 10 human samples. The results showed a clear distinction (Additional file 1: Figure S1A, B). We, respectively, selected the top 3 samples (control: GSM3416675, GSM3416677, and GSM3416679; DCM: GSM3416685, GSM3416689, and GSM3416691) from PCA analysis between the control and DCM group, and performed ChIP-seq analysis by two peak calling software to detect LMNA mutation binding to euchromatin in myocardial tissue. To identify the distribution of LMNA mutation protein binding sites on euchromatin in human cardiac myocytes, we visualized their binding patterns and their chromosomal locations, and found that ChIP peaks were distributed in all chromosomes except for chromosome Y. The top 18 chromosomes contained the most DNA binding sites. Moreover, these DNA binding sites in DCM group were significantly reduced. Meanwhile, we observed that the peak shapes identified by the two software have certain differences. The narrow peaks were identified by MACS, and the broad peaks were identified by SICER (Fig. 1a, b). The narrow peak shape of MACS reflects the binding between LMNA alone and euchromatin, while the broad peak shape of SICER reflects the binding of LAP2α-lamin A/C complexes and euchromatin, which was consistent with previous reports [20, 21]. We also discovered that the density of distributed signal in DCM at low levels compared with control in gene body (from the transcription start site (TSS) to the transcription end site (TES) (Fig. 1c).

### Function enrichment analysis of differential binding loci of LMNA ChIP-seq over euchromatin

**Fig. 2:**
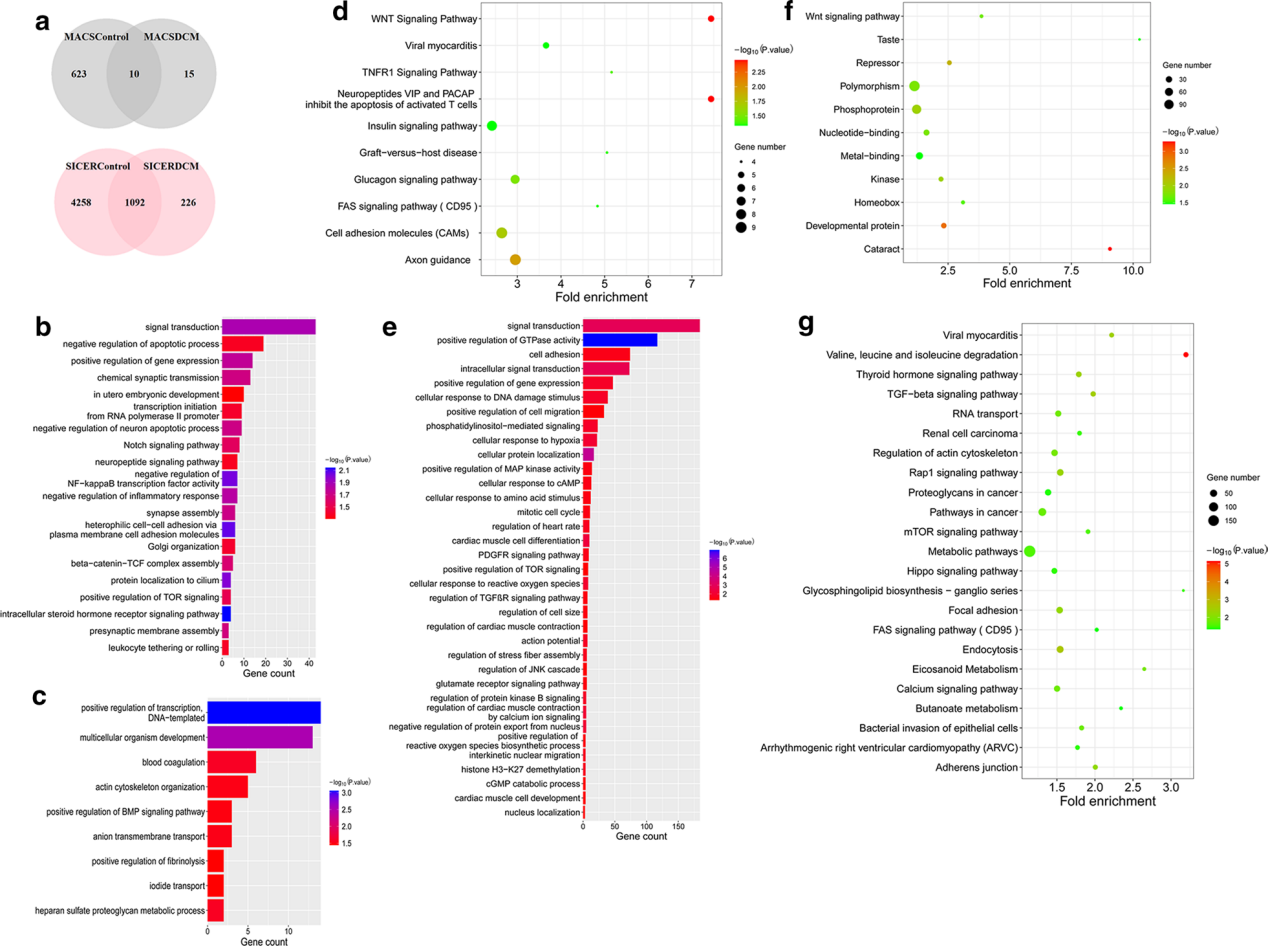
Identification of ChIP-seq differential binding genes, GO annotation, and pathway analysis. **a** Venn diagram indicating the overlap from MACS and SICER between control and DCM samples, respectively. **b-d** Biological process GO terms of differential target genes. **b** LMNA differential target genes in normal tissues. **c** Differential target genes of LAP2α-lamin A/C complexes in DCM. **d** Differential target genes of LAP2α-lamin A/C complexes in control samples. **e–g** Pathway analysis of differential target genes. The color of the circle represents the p value, and the size of the circle refers to the number of genes enriched in the same entry. **e** LMNA differential target genes in normal tissues. **f** Differential target genes of LAP2α-lamin A/C complexes in DCM. **g** Differential target genes of LAP2α-lamin A/C complexes in control samples

We, respectively, intersected the different binding genes across the selected 3 group samples (Additional file 1: Figure S2A). Venn diagram indicated the intersection between the control and the DCM (Fig. 2a). Then, the unique genes were chosen to perform GO and pathway analysis with *p* < 0.05. For the binding between LMNA and euchromatin (MACS), the enrichment results of control group (MACS) in GO biological process (BP) analysis showed that signal transduction and positive regulation of gene expression were involved (Fig. 2b). As to DAVID pathway analysis, some signaling pathways including WNT signaling pathway, FAS signaling pathway (CD95), and TNFR1 signaling pathway were found (Fig. 2e). However, we could not obtain the enrichment results from the limited 15 unique genes in DCM (MACS).

For the regulation of nucleoplasmic LAP2α-lamin A/C complexes (SICER) on euchromatin, the selected 3000 unique genes of control and 226 unique genes of DCM were processed separately via GO and pathway functional analysis. We acquired the annotated results of the 3000 control unique genes (SICER); it contains several biological processes, including intracellular signal transduction, positive regulation of GTPase activity, cellular protein localization, regulation of cardiac muscle contraction, cardiac muscle cell development, cellular response to hypoxia, and oxygen species, regulation of JNK cascade (Fig. 2d). A total of 23 pathways were enriched, and we also obtained the corresponding annotation in signaling transduction, like mTOR signaling pathway and TGF-beta signaling pathway (Fig. 2g). According to the results of BP analysis in DCM (SICER), the enrichment biological processes are shown in Fig. 2c. For the results of pathway annotation, WNT signaling pathway was found through analyzing the DCM target genes (Fig. 2f). The unique genes in both groups and genes associated with functional enrichment mentioned above were collected in Additional file 2: Table Sheets 1–10.

### Identification and visualization of the differential expressed genes (DEGs) through RNA-seq and ChIP-seq analysis

To deeply study the differential genes over euchromatin in LMNA mutation-associated DCM, we selected the genes from RNA-seq data consistent with the differential binding genes of ChIP-seq to perform PCA, and the PCA results also showed distinct clustering at the transcriptome level (Additional file 1: Figure S2B). Furthermore, the heatmap confirmed that the differential target genes of LMNA alone (MACS) and LAP2α-lamin A/C complexes (SICER) according to ChIP-seq results were also shown significant differences in RNA-seq analysis between control and DCM (Additional file 1: Figures S2C,a and S2C,b).

**Fig. 3:**
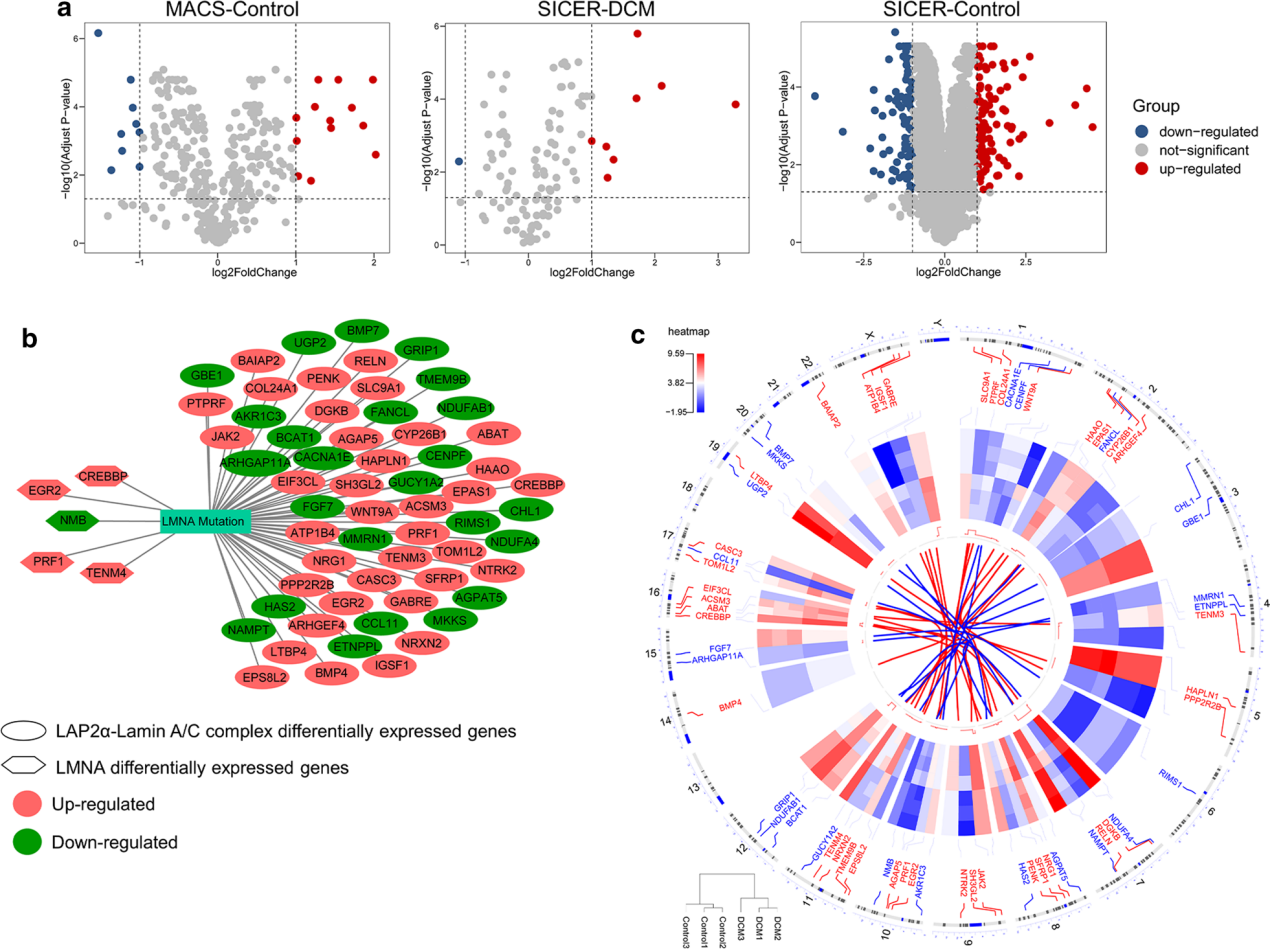
Identification and visualization of the dysregulated binding genes in RNA-seq. **a** Volcano plots for the RNA-seq normalized transcript data consistent with ChIP-seq genes in control and DCM. Red, significantly up-regulated genes; blue, significantly down-regulated genes; gray, no significant change. **b** Possible LMNA regulatory and co-expression networks. Hexagons and ellipses, respectively, represent candidate genes of LMNA and LAP2α-lamin A/C complexes. Pink nodes indicate the up-regulated candidate genes, and the down-regulated candidate genes are shown by green nodes. **c** Circular visualization of connectivity, expression patterns, and chromosomal positions of the candidate genes between functional analysis and DEGs. The outermost circle represents chromosome localization information; inner lines coming from these genes point to their specific chromosomal locations. The down-regulated and up-regulated genes according to adjusted P values are labeled in blue and red and connected with blue and red lines in the center of circles. Expression patterns of candidate genes are represented in the inner circular heatmaps. Blue indicates downregulation and red represents the up-regulation gene. Plots were generated using the Circos visualization tool by OmicCircos package in R

To further confirm the differential binding genes obtained from ChIP-seq, we detected the DEGs in RNA-seq data. The Volcano plot, respectively, showed DEGs in control and DCM, according to criteria of *p* < 0.05 and log_2_FC > 1.0. For target genes associated LMNA alone with euchromatin (MACS), we identified a total of 22 significant genes in control samples (MACS) (Fig. 3a). In order to further discover the genes that are the most related to DCM caused by LMNA mutation in euchromatin, we integrated differential expressed genes, and the genes involved in the functional enrichment analysis mentioned above (Fig. 2b–g, Additional file 2: Table Sheets 4–10) and 5 important genes were selected as candidate genes.

For the target genes of nucleoplasmic LAP2α-lamin A/C complexes in control (SICER), we also identified 183 genes, of which 85 were up-regulated and 98 were down-regulated (Fig. 3a). Similar to the above results, we obtained 60 candidate genes. The binding genes of nucleoplasmic LAP2α-lamin A/C complexes in DCM (SICER), 9 DEGs were displayed (Fig. 3a). However, there was no overlap gene between DEGs analysis and functional enrichment analysis. Here, we noticed that even though there were unique genes in the DCM groups through the ChIP-seq, the candidate genes were not obtained because the unique binding genes to euchromatin in DCM were not changed by RNA-seq analysis. In the final phase, we constructed the interaction network between LMNA mutation and its candidate genes through Cytoscape database. The correlation network revealed that LMNA mutation is mainly associated with 65 candidate genes. Figure 3b shows possible LMNA regulatory and co-expression networks. Four genes were up-regulated, while 1 was down-regulated in 5 candidate genes identified by MACS. Among the 60 genes identified by SICER, 24 of them were up-regulated, while 36 were down-regulated. We selected 65 candidate genes to visualize their expression patterns and chromosome locations, and found that chromosomes 1, 2, 7, 8, 10, 11, and 16 contained the most candidate genes (Fig. 3c).

### Identification of key genes associated LMNA alone or LAP2α-lamin A/C complexes with euchromatin in DCM

**Fig. 4:**
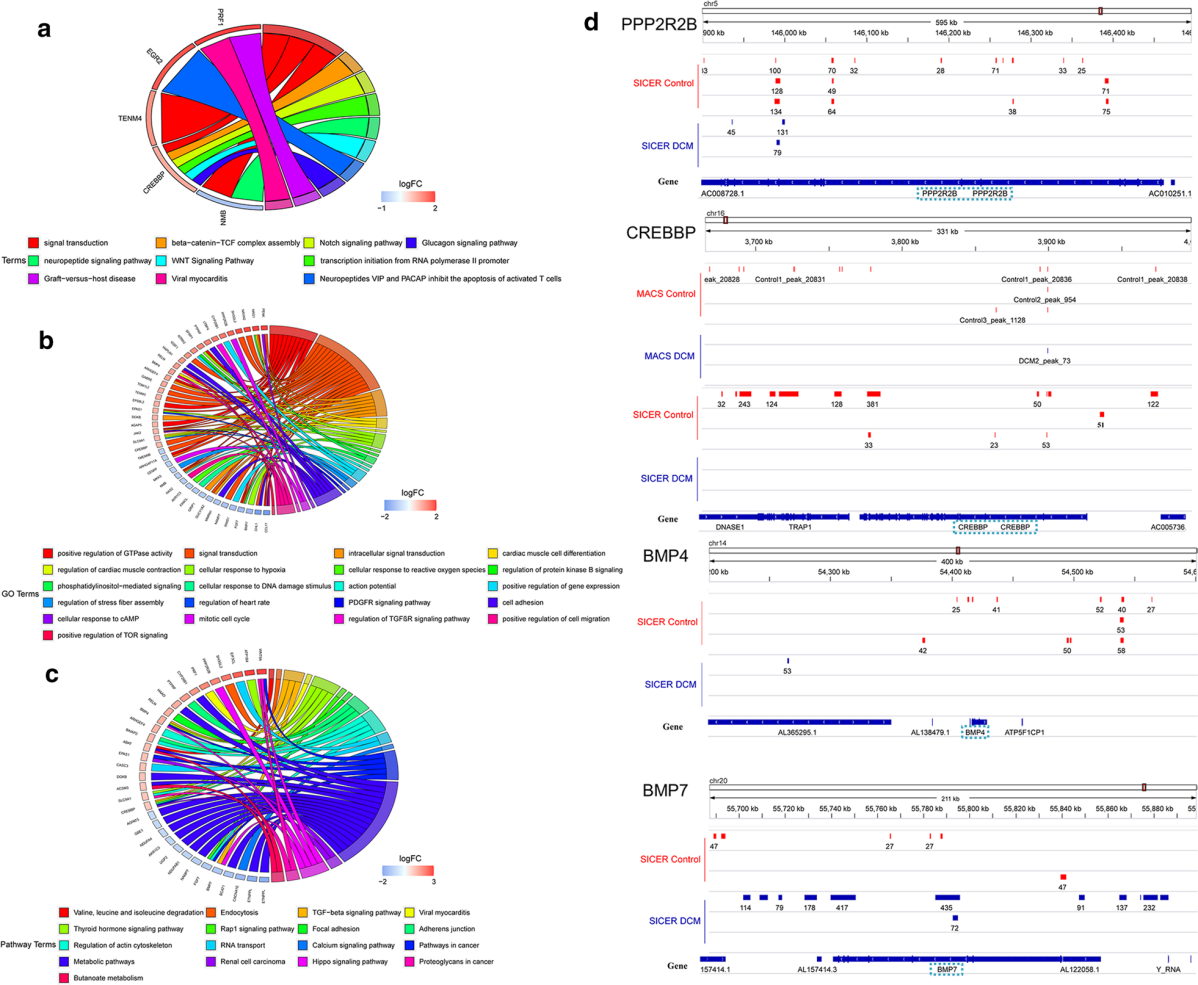
Functional enrichment analysis of candidate genes and visualization of the signal distribution of key genes. **a** Chord plot depicting the relationship between genes obtained from MACS and GO terms of biological process or pathway enrichment. **b** Chord plot showing the relationship between genes identified from SICER and GO terms of biological process. **c** Chord plot indicating the relationship between genes from SICER and pathway analysis. **d** Integrative Genomics Viewer tracks of PPP2R2B, BMP4, CREBBP, and BMP7 (hg19). Integrated Genomic Viewer browser image showing signals of the hub genes in Control (red) and DCM (blue)

Sixty-five candidate genes were chosen to perform GO and KEGG analysis. For the candidate genes measured by MACS (LMNA alone bind to euchromatin), we detected enrichment in several biological process GO terms such as signal transduction, beta-catenin–TCF complex assembly, transcription initiation from RNA polymerase II promoter, and Notch pathway (Fig. 4a). In terms of pathway annotation, WNT pathway was the most significantly enriched result, which showed a high correlation with CREB binding protein (CREBBP). It indicated that CREBBP was the key gene. We visualized the distribution of lamin A/C-binding sites in euchromatin by Integrated Genomic Viewer browser (IGV) (Fig. 4d). The signals of CREBBP in DCM were significantly lower than control, which suggested that the binding between lamin A/C and DNA on euchromatin was impaired in the LMNA mutant.

For the biological process of the candidate genes identified by SICER (LAP2α-lamin A/C complexes bind to euchromatin), signal transduction and cardiac muscle cell differentiation are shown in Fig. 4b. As to pathway analysis of the candidate genes identified by SICER, we observed that TGF-beta pathway was mostly associated with these genes (Fig. 4c). Here, we selected 4 genes (CREBBP, BMP4, BMP7, and PPP2R2B) as key genes according to TGF-beta pathway and consulted literature, which showed a high correlation with the study. The 4 key genes (PPP2R2B, BMP4, CREBBP, and BMP7) were distributed in chromosomes 5, 14, 16, and 20 (Fig. 3c), and we identified the signal distribution of the 4 key binding sites (Fig. 4d). The signal strengths of these genes were reduced in the DCM compared to the control group, except for BMP7.

### De novo motif analysis in euchromatin

**Fig. 5:**
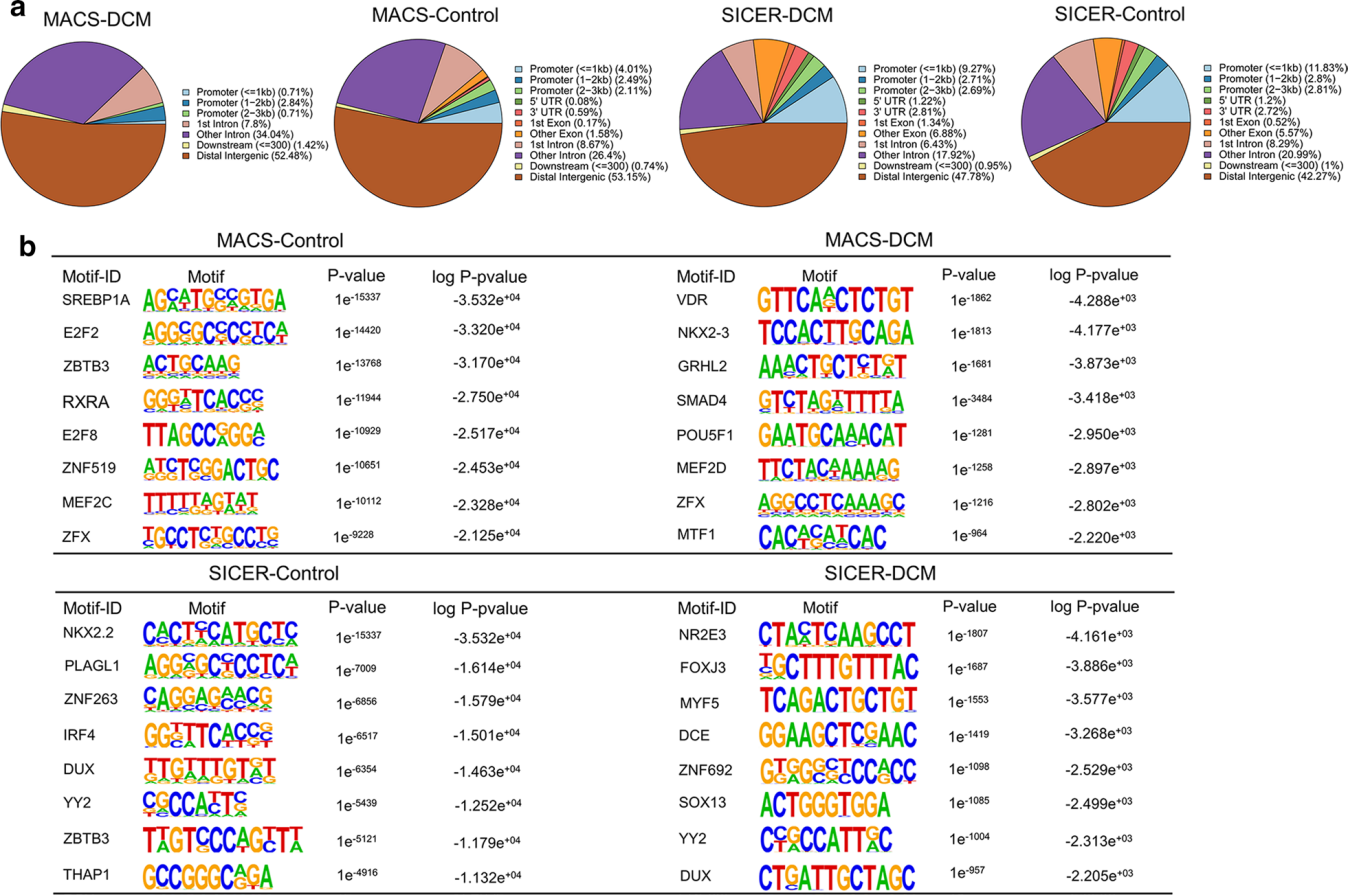
TFBS motif enrichment analysis. **a** Pie chart illustrating the distribution of peak locations, including promoter, 5′UTR, and 3′UTR, exon, intron, intergenic in the genome. **b** TFBS motifs. Logo showing the top 8 motifs identified using MACS and HOMER software

We, respectively, visualized the genomic locations of binding sites of LMNA alone (MACS) and LAP2α-lamin A/C complexes (SICER) using the ChIPseeker by R package. We found that the majority of binding sites of LMNA and LAP2α-lamin A/C complexes were located in distal intergenic regions, followed by introns, promoters, and to a lesser degree in exons, 3′ UTRs and 5′ UTRs. Compared with LMNA binding sites, the locations of intergenic regions were significantly reduced in binding sites of LAP2α-lamin A/C complexes, meanwhile, the locations of promoters were obviously increased. Furthermore, these pie charts also illustrated that the promoter regions of binding sites in both LMNA and LAP2α-lamin A/C complexes in DCM were significantly lower than control groups (Fig. 5a).

Based on the above analysis of genomic locations of LMNA and LAP2α-lamin A/C complex binding sites, we found that LMNA mutation influenced transcription factor binding at the promoter regions in euchromatin (Figs. 1c and 5a). We further performed the de novo transcription factor binding sites (TFBSs) analysis to identify TF binding motifs in the LMNA and LAP2α-lamin A/C complex binding regions located at the promoters in cardiac myocyte euchromatin. Motifs were sorted along with the corresponding p values, the top 8 predicted motifs are shown in Fig. 5b. The results demonstrated that the enriched motifs were extraordinary differences. SREBP1A motif (a novel lamin A interactor and the coactivator of CREBBP) was discovered in the control group of MACS (LMNA alone bind to euchromatin). Meanwhile, IRF4 (a factor associated with CREBBP) and TCF3 (a β-catenin-binding effector to repress of WNT signaling) were unique to control of SICER (LAP2α-lamin A/C complexes bind to euchromatin), which is in accord with the previous research [22–25]. A complete list of the enriched TFBSs in the LMNA and LAP2α-lamin A/C complex binding sites located in the promoter region is shown in Additional file 1: Figures S3 and S4.

## Discussion

DCM caused by LMNA mutations remains one of the most aggressive and lethal heart diseases because of the complexity of LMNA molecule and cellular heterogeneity. The novel insights into the molecular mechanisms underlying LMNA mutation-associated DCM have been reported, and the studies focused on hundreds of chromatin domains in heterochromatin, which are referred to as LADs [9, 20]. However, little is known about LMNA mutation on euchromatin and its role in LMNA mutation-associated DCM. Recently, accumulated evidences showed that lamin A/C also occupied euchromatin regions throughout the nucleoplasm and regulated euchromatin organization and gene expression by direct binding or depending on LAP2α [15]. However, the interaction between LMNA and euchromatin remains unclear in LMNA mutation-associated DCM. In this study, we enrolled 6 qualified datasets from 10 GEO datasets and identified many robust differential binding genes by ChIP-seq technology (Additional file 1: Figures S1A and S1B, Additional file 2: Table Sheets 11–14). We found that the Gene Ontology of the differential binding genes was mainly involved in signal transduction by DAVID Bioinformatics Tool. However, because we could not obtain the enrichment results from the direct binding genes between LMNA and euchromatin (MACS) in DCM group, we further used the clusterProfiler package by R to validate the annotation results. As the same with DAVID enrichment, there were no referential enrichment results (Additional file 1: Figure S5). In addition, enrichment analysis of the identified differential binding genes in some pathways, such as WNT signaling pathway, mTOR pathway, TGF-beta pathway, TNFR1 pathway, and FAS pathway (CD95), is consistent with the previous researches [7, 26–29]. For example, Le Dour C et al. demonstrated that the downregulated WNT/β-catenin pathway in the hearts contributed to the pathophysiology of LMNA cardiomyopathy [28]. Choi et al. confirmed that AKT-mTOR pathway was hyperactivated with cardiomyopathy caused by *LMNA* mutation [27]. Tan CY group indicated that cardiac myocyte-specific expression of pathogenic variations in LMNA, associated with DCM, led to abnormal activation of the TGF-beta pathway in the heart and induction of cardiac dysfunction, and premature death [7, 29]. Several studies have reported that strong activation of the pro-apoptotic TNF and Fas pathways in DCM patients [26].

**Fig. 6:**
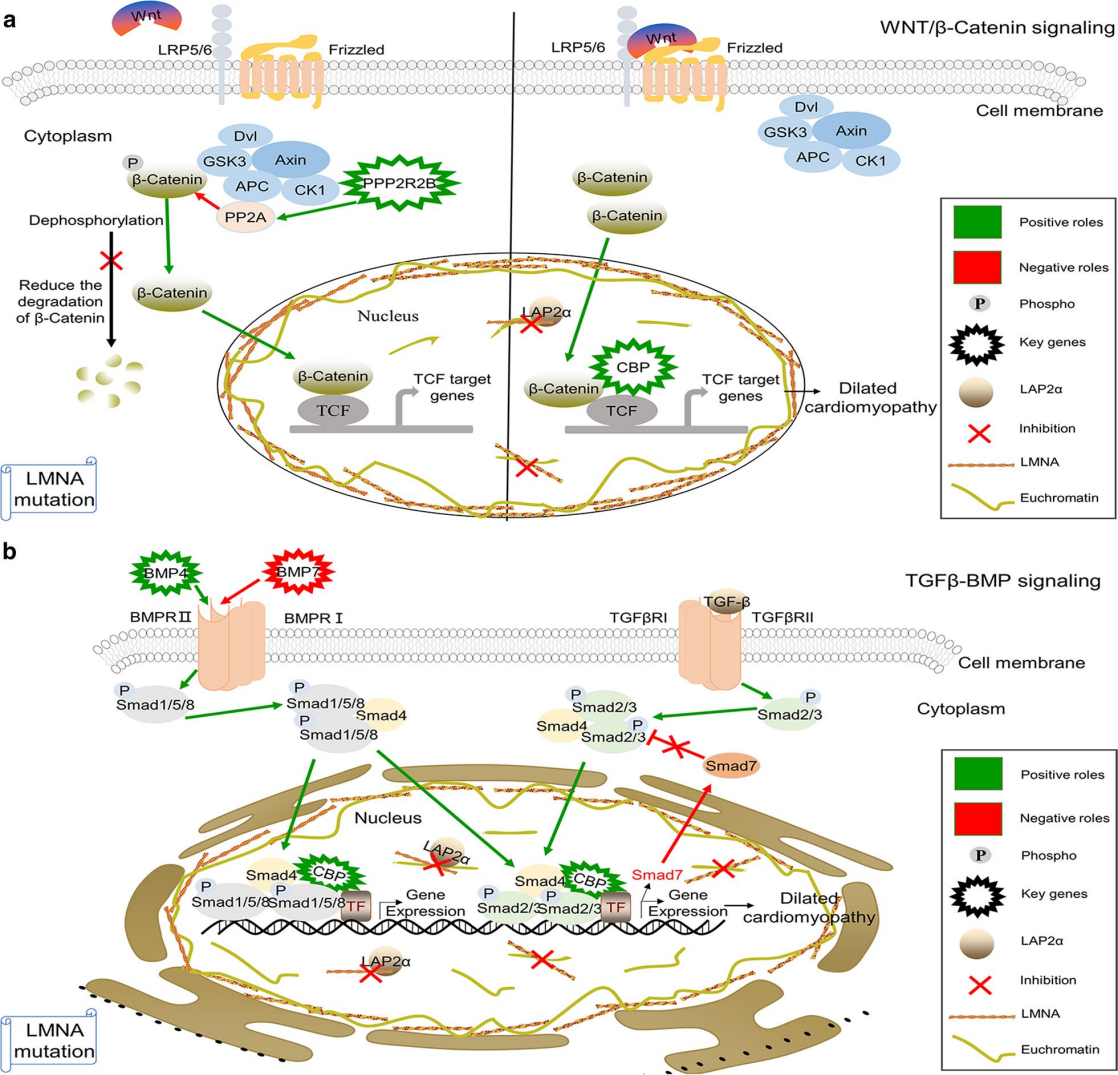
Possible mechanism diagram of key genes in TGFβ-BMP and WNT/β-catenin signal pathway

We further identified the differential binding genes through integrating analysis ChIP-seq and RNA-seq data. For the differential genes associated LMNA alone with euchromatin, we obtained 5 candidate genes (EGR2, NMB, CREBBP, PRF1, and TENM4), of which EGR2 and NMB have been reported to involve in myocardial ischemia and heart failure, respectively [30, 31]. Although there are currently no reports about PRF1 and TENM4 involved in heart disease, we noticed that they may be related to LMNA mutation-associated DCM in our research; therefore, they may be the potential biomarkers of DCM, which needs further verification. CREBBP was demonstrated to exert essential roles in the pathogenesis of DCM [32], which is consistent with our experimental results. In our study, CREBBP was discovered as the only key gene in the control groups, and the TF motif (SREBP1A) which is a novel lamin A interactor and the coactivator of CREBBP also had a significant change between control and DCM groups (Fig. 5b). Meanwhile, CREBBP has been widely reported to take part in WNT/β-catenin pathway [33], but to our knowledge, the role of CREBBP participating in WNT/β-catenin pathway in LMNA mutation-associated DCM was still unclear. From our results, we proposed a possible link among CREBBP, WNT pathway and LMNA mutation-associated DCM, which helped to explain the mechanism of DCM caused by LMNA mutation (Fig. 6a). CREBBP, a β-catenin-interacting protein functions as a coactivator in WNT signaling. Mutations in the LMNA gene may cause LMNA encoded protein to fail to bind to CREBBP DNA on the euchromatin, and our results showed that the expression of CREBBP was increased at the transcript level. We speculated that the increased CREBBP binds to the nuclear β-catenin and TCF, which led to excessive activation of WNT pathway, and promotes changes in transcription mechanism and causes the activation of several target genes, such as c-jun, c-Myc, Fos-Related Antigen-1, and COX2. Ultimately those changes contribute to the pathological phenotype of LMNA mutation-associated DCM. Although we could not obtain the enrichment results from the genes in DCM samples, we still carried out further analysis on its unique genes. We found that the expression of UCN3 gene was dysregulated in DCM patients, which is consistent with previous experimental data [34].

For the regulation of nucleoplasmic LAP2α-lamin A/C complexes, we eventually obtained 60 candidate genes (Additional file 2: Table Sheet 15) in control groups. Some candidate genes have been reported to be related to the development of DCM, such as BMP4, BMP7, CREBBP, and NAMPT [29, 32, 35, 36]. Furthermore, SLC9A1 and CACNA1E have been reported to regulate the development of other heart diseases, such as heart failure and arrhythmia [37, 38]. Based on our integrated analysis results, we considered that some key genes named BMP4, PPP2R2B, BMP7, and CREBBP might play the key roles in LMNA mutation-associated DCM, and they might be used to predict the diagnosis and prognosis of the disease. Similar to the change of CREBBP in the regulation of LMNA alone, there was also a significant change in TF motif (IRF4) related to CREBBP (Fig. 5b); therefore, the role of CREBBP in the pathogenesis of DCM cannot be ignored. Increasing experimental data have been reported that bone morphogenetic protein 4 (BMP4), an important member of the TGF-β superfamily, leads to enhanced autocrine activation of TGF-β-responsive SMAD by regulating the activity of a series of downstream genes, which providing a context for the activation of TGF-β signaling in response to BMP during development [39]. In addition, the abnormality of protein phosphatase 2 regulatory subunit Bbeta (PPP2R2B) has been confirmed to be involved in WNT pathway [40]. At the same time, we noticed that WNT/β-catenin signaling inhibitor TCF3 motif was not found in DCM in the study, which further verified the disorder of WNT/β-catenin pathway (Figs. 5b and Additional file 1: Figure S4). Although these genes have been associated with the pathway mentioned above, how dysregulation of these genes affects LMNA mutation-associated DCM is not understood. Previous research believed that PPP2R2B acts as a WNT signal antagonist, which is contrary to our study results [41]. Ishibashi et al. reported that the up-regulated PPP2R2B activates WNT pathway through promoting the dephosphorylation of β-catenin by PP2A [40]; our results were consistent with them (Fig. 6a). In this study, we speculated two possible mechanisms to explain how these genes that are regulated by the LAP2α-lamin A/C complexes on euchromatin lead to LMNA mutation-associated DCM. The LMNA mutations might be accompanied by the LAP2α-lamin A/C complexes not binding to BMP4 and CREBBP DNA on euchromatin and increasing the transcriptomic level of BMP4 and CREBBP. At the same time, the BMP4 ligand and the serine/threonine kinase receptor II (BMP receptor II, BMPR II) on the cell membrane excessively interacts to form a ligand receptor binary complex. The BMPR II can hyperactivate the type I receptor (BMPR I), and the activated BMPR I further phosphorylates the Smad protein (Smad1, Smad5, and Smad8) to promote the detachment of Smad molecules from the cell membrane receptor. The Smad1/5/8 protein forms heteromeric complexes with Smad4 (common Smad, Co-Smad) and translocates to the nucleus. In the nucleus, the Smad multiplex complex regulates the transcription of some specific target genes with the participation of CREBBP, which contributes to hyperactivate the TGF-β pathway and may further lead to LMNA mutation-associated DCM. Interestingly, we found that the dysregulation of bone morphogenetic protein 7 (BMP7) exerted similar effects with BMP4 on the regulation of TGF-β pathway. We discovered the increased binding between LAP2α-lamin A/C complexes and BMP7 DNA and decreased expression of BMP7 mRNA in DCM. BMP7 binds to the BMPR II, promoting phosphorylation of Smad1/5/8, which initiates downstream signal transduction pathways. Phosphorylated Smad1/5/8 binds to the Smad4 molecule in the cytoplasm and translocates to the nucleus to regulate the expression of some specific genes. The reduction of BMP7 expression level contributed to reducing Smad7 (an intracellular antagonist of TGF-β/Smad signaling) expression and the inhibitory effect on Smad3 DNA binding. Therefore, phosphorylated Smad2/3 and the heteromeric complexes were promoted, which exerted excessive positive regulation of TGF-β pathway with the participation of CREBBP (Fig. 6b). However, we also obtained 4 genes by GO and DAVID analysis in DCM groups, including TRABD2B, TLE3, CTNND2, and ROR2, but these genes were no significant difference at the transcriptomic level. However, previous reports have suggested that ROR2 played a distinctive role in signal transduction in human cardiomyocyte development [42]. Although we predicted the detailed mechanisms on LMNA mutation-associated DCM, the results also need to further be validated by experiments. We also notice other limitations in our study; we could not obtain the enrichment results from the limited 15 unique genes in DCM (MACS). The three samples have different mutation sites, which may filter out some specific and unique genes related to distinct mutation sites. All the above problems need to be further solved in future studies.

According to the integrated results of differential expressed genes, GO, and pathway annotation, we posit that these differential binding genes in euchromatin are closely associated with dysregulated WNT and TGF-β pathways responsible for LMNA mutation-associated DCM.

## Conclusions

LMNA mutations can affect the binding between LMNA or LAP2α-laminA/C complexes and euchromatin DNA. The disorder binding may cause DCM through the changes of CREBBP, PPP2R2B, BMP4, BMP7 expressions, and the dysregulation of WNT/β-catenin or TGFβ-BMP pathways. Our findings demonstrate the significance of WNT/β-catenin or TGFβ-BMP pathways and provide a theoretical basis that CREBBP, PPP2R2B, BMP4, and BMP7 serve as the potential biomarkers in LMNA mutation-associated DCM. By the proposed molecular pathogenesis mechanism, we provided valuable insights into the pathogenesis of LMNA mutation-induced DCM, which provides clinical value for its treatment.

## Methods

### The overview of the study

**Fig. 7:**
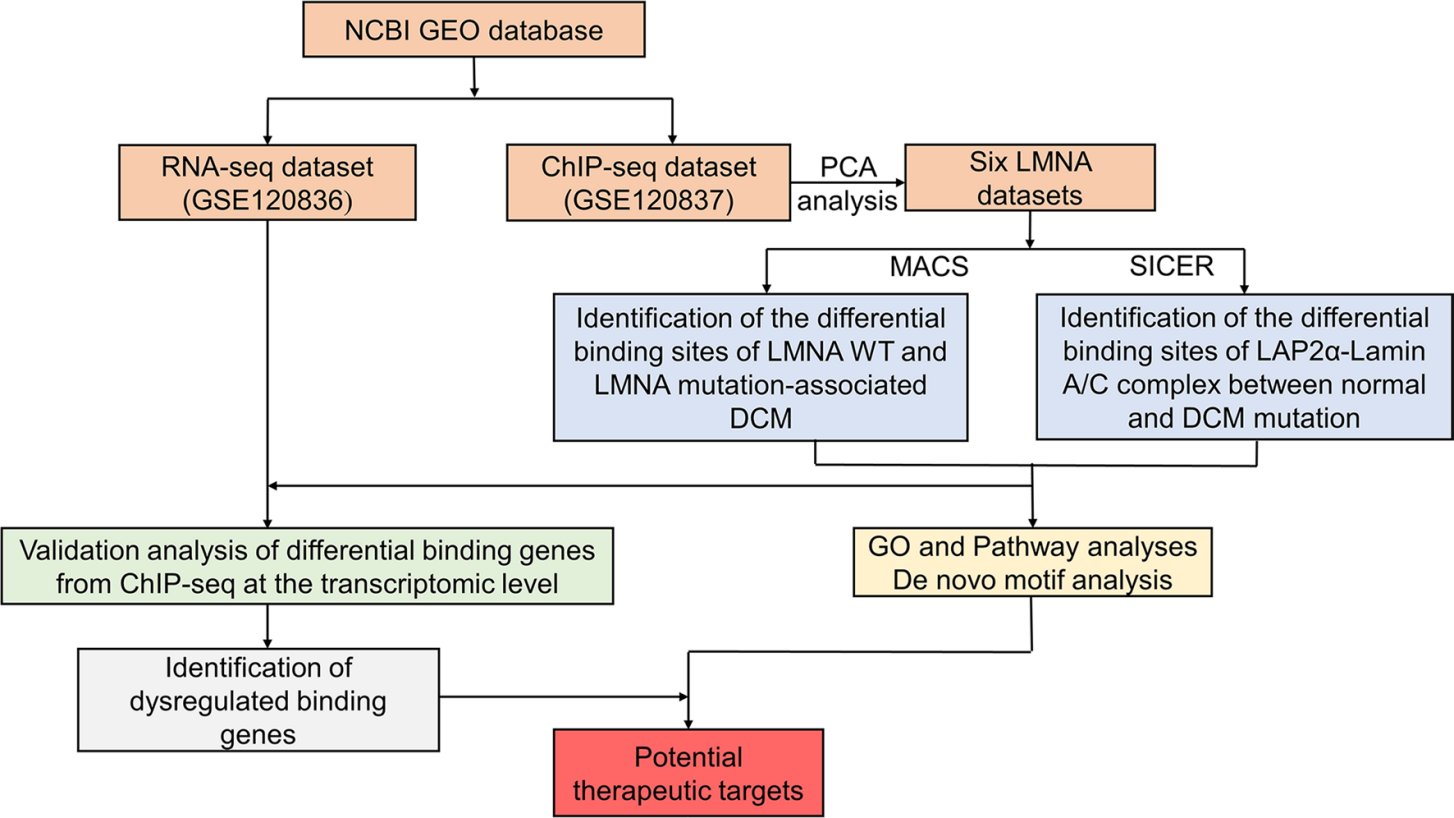
Study flowchart of presenting the study design. NCBI, National Center for Biotechnology Information

In order to make this study more comprehensible, we draw a flowchart. Through the analysis of ChIP-Seq and RNA-seq data, some key differential expressed genes whose binding sites on euchromatin were changed by LMNA mutations were obtained, and these genes were deeply analyzed to further clarify the pathogenesis of DCM caused by LMNA mutations. It is visualized in Fig. 7.

### Acquisition of gene datasets

All datasets were downloaded from the GEO database. RNA-seq datasets were obtained from GSE120836, and ChIP-seq datasets were downloaded from GSE120837. ChIP-seq datasets were from 5 DCM patients and 5 normal persons. RNA-seq datasets were derived from 3 groups of myocardial tissues.

### ChIP-seq analysis

The quality of the ChIP-seq dataset was inspected using FastQC. Reads were aligned to human reference genome hg19 (GRCh37) using Bowtie 2 [43]. Artifactual read duplicates were removed prior to further analysis. The deduplicated reads were indexed and sorted using SAMtools for further processing. MACS2 [44] was run with the p value of 0.01 to perform the peak calling against the corresponding input sample to identify the LMNA alone. BamCoverage was used to convert sorted BAM files into BW format. Principle component analysis calculated and drawn correlation by deeptools [45]. We obtained the genomic annotation files using the ChIPseeker package in R/Bioconductor [46]. We performed identification of the enriched sequence motifs within ChIP-Seq peak regions using HOMER software package [47]. Integrated Genomic Viewer browser was used to visualize peaks between different groups. We integrated the overlapping gene symbol names from the annotation file for further differential binding genes analysis. Overlapping gene symbols were uploaded into the DAVID web (https://david.ncifcrf.gov/). Gene Ontology terms and pathway enrichment analysis with parameter *p* < 0.05 were performed with DAVID database and visualized by ggplot2 (R package). Meanwhile, we used SICER software [48] with FDR = 0.001 and other default parameters to identify the nucleoplasmic LAP2α-lamin A/C complexes. The other procedures are the same as those of MACS, including the following steps.

### RNA-seq analysis

We integrated differential binding genes (*N* = 3) from ChIP-seq with the corresponding RNA-seq data, respectively. For the captured three groups transcriptome genes, we performed the PCA of transcript levels using the Plot3D package in R. We utilized the heatmap.2 function in the package of ggplots to visualize the transcription expression levels of differential binding genes obtained from ChIP-seq.

### Analysis of dysregulated binding genes

We used R package of Limma [49] to find and screen the DEGs with the parameters *p* value < 0.05 and log_2_fold change (log_2_FC) > 1. We obtained the dysregulated binding genes via the overlap between differential binding genes from ChIP-seq and DEGs of RNA-seq. Dysregulated binding genes were visualized by Cytoscape [50], GOplot, and OmicCircos.

## Supplementary information

**Additional file 1**. **Figure S1**. Principal component analysis of 10 samples. **Figure S2**. Identify the overlapping binding genes from ChIP-seq and RNA-seq. **Figure S3**. Enriched transcription factor binding site motifs identified using MACS. **Figure S4**. Transcription factor binding site motifs identified using SICER. **Figure S5**. GO and pathway analysis by ClusterProfier package.

**Additional file 2**. **Table S, Sheets 1**. MACS control unique genes. **Table S, Sheets 2**. MACS DCM unique genes. **Table S, Sheets 3**. SICER control unique genes. **Table S, Sheets 4**. SICER DCM unique genes. **Table S, Sheets 5**. MACS control GO genes. **Table S, Sheets 6**. MACS control pathway genes. **Table S, Sheets 7**. SICER control GO genes. **Table S, Sheets 8**. SICER control pathway genes. **Table S, Sheets 9**. SICER DCM GO genes. **Table S, Sheets 10**. SICER DCM pathway genes. **Table S, Sheets 11**. MACS control overlap genes. **Table S, Sheets 12**. MACS DCM overlap genes. **Table S, Sheets 13**. SICER control overlap genes. **Table S, Sheets 14**. SICER DCM overlap genes. **Table S, Sheets 15**. 60 SICER candidate genes.

## Data Availability

The ChIP-seq data and RNA-seq in this study are available from the Gene Expression Omnibus (GEO, http://www.ncbi.nlm.nih.gov/geo/). The accession number of RNA-seq and ChIP-seq data is GSE120836 and GSE120837, respectively.

## Abbreviations

DCM: Dilated cardiomyopathy
LADs: Lamina-associated domains
LAP2α: Lamina-associated polypeptide 2 alpha
LMNA: Lamin A/C
MAPK pathway: Mitogen-activated protein kinases pathway
mTORC1 pathway: Mammalian target of rapamycin complex 1 pathway
INM: Inner nuclear membrane
ChIP-seq: Chromatin immunoprecipitation sequencing
RNA-seq: RNA sequencing
GEO: Gene Expression Omnibus
GO: Gene Ontology
PCA: Principle component analysis
TSS: Transcription start site
TES: Transcription end sites
BP: Biological process
DEGs: Differential expressed genes
CREBBP: CREB binding protein
IGV: Integrated Genomic Viewer browser
TFBSs: Transcription factor binding sites
BMP4: Bone morphogenetic protein 4
PPP2R2B: Protein phosphatase 2 regulatory subunit Bbeta
BMPR II: BMP receptor II
BMP7: Bone morphogenetic protein 7
NCBI: National Center for Biotechnology Information
