# Supplementary material for: Integrated analysis reveals the alterations that LMNA interacts with euchromatin in LMNA mutation-associated dilated cardiomyopathy
Source: Clin Epigenetics. 2021 Jan 6;13:3. doi: 10.1186/s13148-020-00996-1 (PMC7788725; doi:10.1186/s13148-020-00996-1)
Supplement: Supplementary file 1 — Additional file 1. Figure S1. Principal component analysis of 10 samples. Figure S2. Identify the overlapping binding genes from ChIP-seq and RNA-seq. Figure S3. Enriched transcription factor binding site motifs identified using MACS. Figure S4. Transcription factor binding site motifs identified using SICER. Figure S5. GO and pathway analysis by ClusterProfier package. [file 13148_2020_996_MOESM1_ESM.pdf]

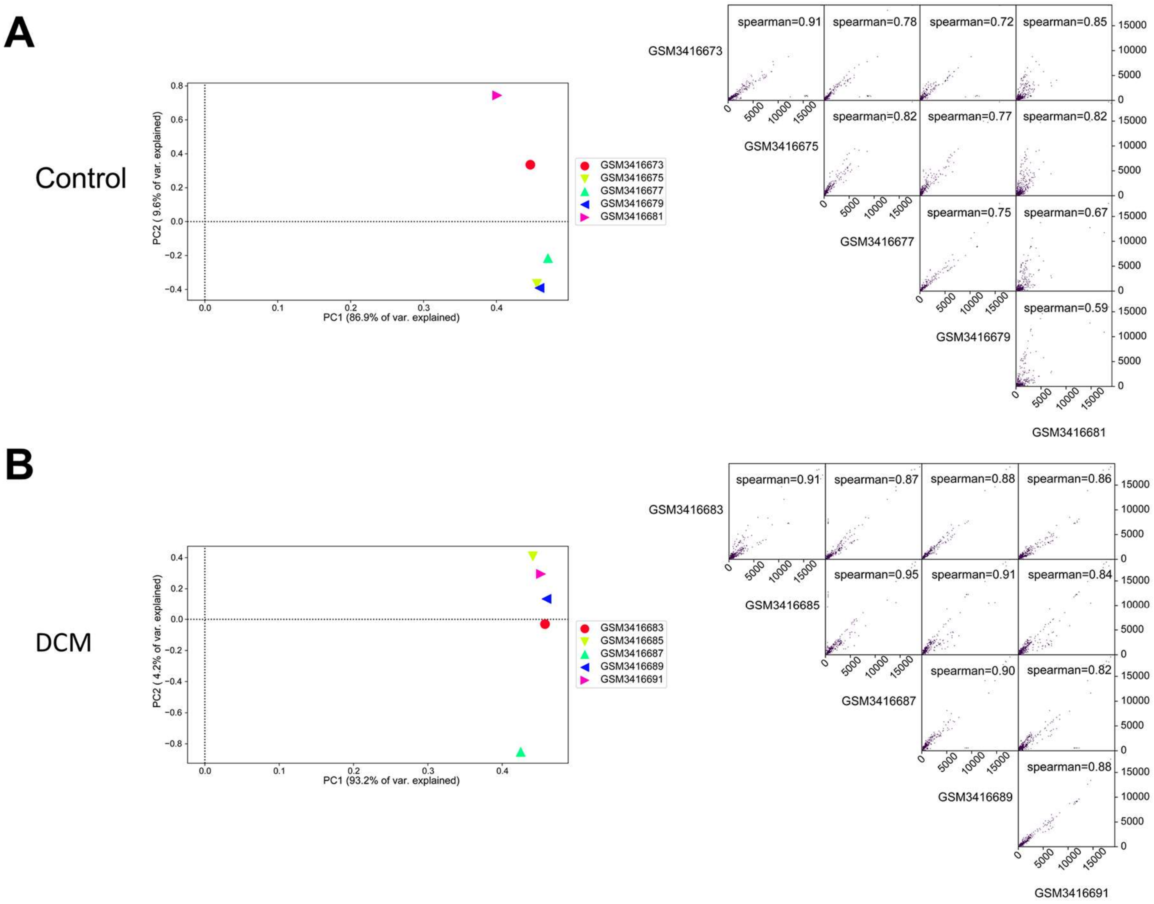

Figure S1. Principal component analysis of 10 samples. **A** Control group; **B** DCM group.

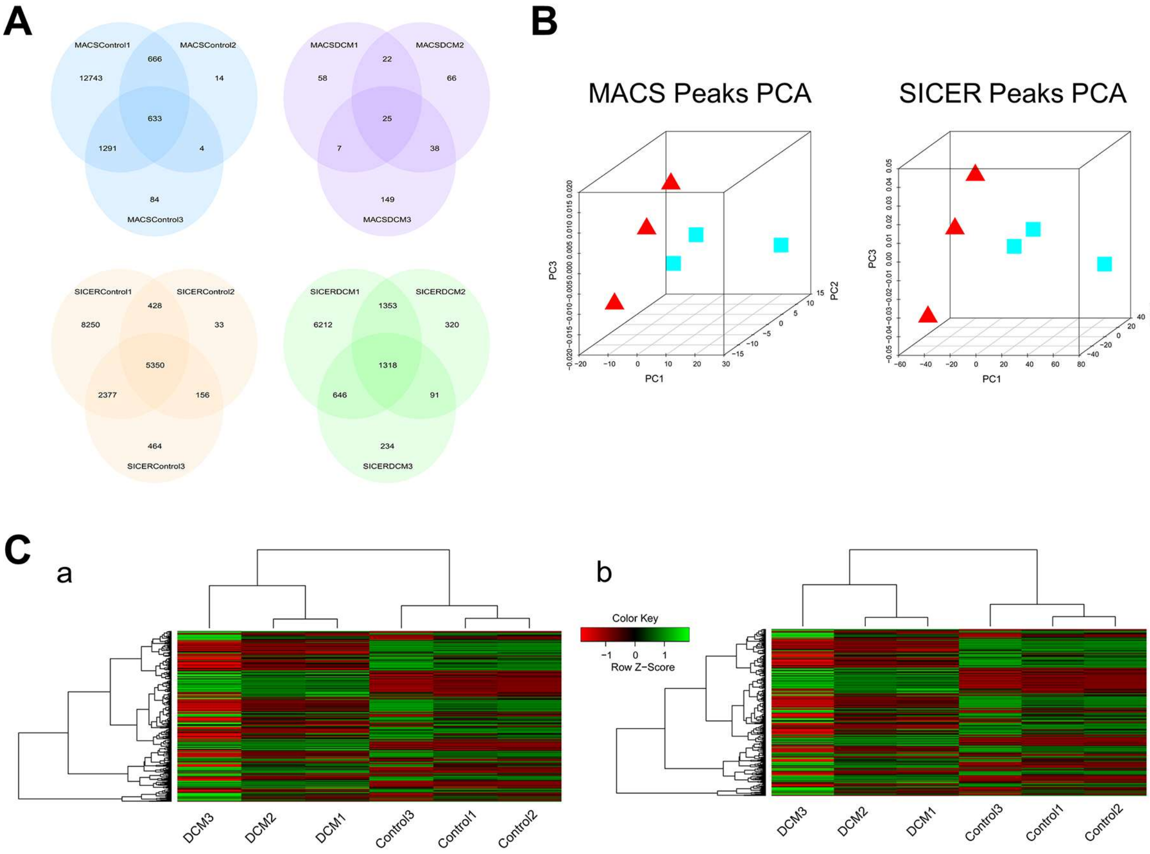

Figure S2. Identify the overlapping binding genes from ChIP-seq and RNA-seq. **A** Venn diagram showing overlaps of the differential binding genes across selected 3 same group samples, respectively. **B** Principle component analysis of the normalized RNA-seq data that consistent with ChIP-seq target genes showing distinct clustering of 3 control and 3 DCM samples. Red, DCM samples; blue, control samples. **C** Heatmap showing the hierarchical clustering of RNA-seq normalized transcript data consistent with ChIP-seq genes based on control and DCM samples. The color from blue to red shows a trend from low expression to high expression. **a** LMNA target genes in both DCM and control tissues. **b** target genes of LAP2α-lamin A/C complexes in both DCM and control samples.

MACS-Control

Motif-ID	Motif	P-value	log P-pvalue
EOMES	GAAGTGACGG	1e-8665	-1.995e+04
ZBTB7B	AGCCCATC	1e-8048	-1.853e+04
TCF12	CGGCTGGCCGGG	1e-7777	-1.791e+04
PRDM1	TTTCACCGTG	1e-7276	-1.675e+04
GLIS3	CCTCCCGG	1e-7005	-1.613e+04
GFI1B	AAGCGGAGAT	1e-6889	-1.586e+04
RHOXF1	GGCGGATCACGA	1e-5758	-1.326e+04
YY1	CGAGATCTCGGC	1e-5709	-1.315e+04
THRb	ACGAGGTC	1e-5502	-1.267e+04
KLF12	ACTACGCC	1e-3651	-8.408e+03
ZFP410	ATGGAATGGAAT	1e-3500	-8.061e+03
STAT3	CTTCCCAGATGG	1e-3249	-7.482e+03
OBOX1	GATAATTCCGTT	1e-2979	-6.859e+03
VDR	GTTCAACTCTGT	1e-2819	-6.491e+03
BMYB	AAACTGCTCTAT	1e-2785	-6.414e+03
TCF7	TTTTGATGTG	1e-2414	-5.560e+03
SOX5	AAAGAGTGTT	1e-2229	-5.133e+03
PROX1	AGAAGGCTTC	1e-1977	-4.553e+03
GM397	ACACACACACAC	1e-1821	-4.195e+03
ZNF692	GTGGGGCCTACC	1e-1754	-4.040e+03
NFIL3	GTTTAGGTAA	1e-1418	-3.265e+03
SMAD3	CAAGCCAGCCAA	1e-365	-8.426e+02
PSE	ACCCTAACCCTA	1e-88	-2.033e+02

MACS-DCM

Motif-ID	Motif	P-value	log P-pvalue
DCE	TGAGAATGCTTC	1e-1979	-4.558e+03
SOX8	GTGAGTTGAA	1e-942	-2.169e+03
GATA5	GGAGATAT	1e-886	-2.042e+03
TFAP2B	GCCTATGGTG	1e-884	-2.036e+03
HDX	CGAAATCCTC	1e-678	-1.562e+03
DCE	GTAGAATC	1e-605	-1.393e+03
TCF3	TCACAAAG	1e-587	-1.354e+03
HOXB13	TTTATGCG	1e-549	-1.264e+03
TCF7	ATCAAAGGGA	1e-513	-1.181e+03
T	ATCACCTAGGTG	1e-484	-1.116e+03
GFI1B	ATCAGTGCAGAG	1e-470	-1.083e+03
GATA3	TAGACAAGAGTT	1e-459	-1.057e+03
ZFX	ATAGGCCT	1e-400	-9.233e+02
ATF1	GATATGTCAC	1e-284	-6.549e+02
PRDM9	CACCACCATCAC	1e-117	-2.709e+02
NKX2.2	TCCACTCCAC	1e-69	-1.599e+02
PSE	CTAACCCTAACC	1e-57	-1.317e+02
RUNX2	CACCACAC	1e-0	-2.143e+00

Figure S3. Enriched transcription factor binding site motifs identified using MACS.

SICER-Control				SICER-DCM			
Motif-ID	Motif	P-value	log P-pvalue	Motif-ID	Motif	P-value	log P-pvalue
IRF5	ATCGAGAC	$1e^{-4511}$	$-1.039e^{+04}$	HNF6	GCAGTTCGAT	$1e^{-949}$	$-2.186e^{+03}$
ZFP105	GTAATTTATTGA	$1e^{-4200}$	$-9.672e^{+03}$	ZNF768	GCAGCGAGGCTG	$1e^{-897}$	$-2.068e^{+03}$
LHX8	TCCCGGCTAATA	$1e^{-4180}$	$-9.625e^{+03}$	ZAC1	TCCCGGCTAATA	$1e^{-804}$	$-1.852e^{+03}$
SMAD4	AGACGGGG	$1e^{-4017}$	$-9.251e^{+03}$	ZFX	AGGCAGGCCTGC	$1e^{-775}$	$-1.785e^{+03}$
TCF3	GTTTGATATCAG	$1e^{-3969}$	$-9.140e^{+03}$	MAFA	AAGGCAGCAG	$1e^{-646}$	$-1.488e^{+03}$
MAX-MYC	ACCTCGTGAT	$1e^{-3951}$	$-9.100e^{+03}$	THAP1	TCTGGGGGCAGG	$1e^{-632}$	$-1.456e^{+03}$
SRF	TTTTTTCTAAGG	$1e^{-3785}$	$-8.716e^{+03}$	EGR1	GGGTCCCACGCC	$1e^{-580}$	$-1.337e^{+03}$
LRF	CGTCCCTTGT	$1e^{-3772}$	$-8.687e^{+03}$	RUNX	CCACCACAGC	$1e^{-562}$	$-1.295e^{+03}$
CEBP	CTGCTCAACGAA	$1e^{-3556}$	$-8.189e^{+03}$	SMAD4	TCTACGTCTG	$1e^{-544}$	$-1.253e^{+03}$
SOX11	TTGTCAATTTTG	$1e^{-3482}$	$-8.019e^{+03}$	BCL6B	CGCCCCTCCC	$1e^{-523}$	$-1.206e^{+03}$
ZFP187	TATACACCAATA	$1e^{-3371}$	$-7.762e^{+03}$	NFYB	TACACCAATCAG	$1e^{-511}$	$-1.177e^{+03}$
HIC1	GTTGGTAGTTCT	$1e^{-3308}$	$-7.619e^{+03}$	SIX6	TATATCCCGCAC	$1e^{-495}$	$-1.141e^{+03}$
SF1	TTGCCCTTGC	$1e^{-3284}$	$-7.562e^{+03}$	GATA	GATAATATCC	$1e^{-491}$	$-1.131e^{+03}$
ZBTB3	GAAAACTGGC	$1e^{-3216}$	$-7.406e^{+03}$	RXRA	TTCACATAGTTC	$1e^{-443}$	$-1.020e^{+03}$
NFkB	GAAGGATTCCCT	$1e^{-2971}$	$-6.841e^{+03}$	CRX	GAAGTTTAGAGA	$1e^{-395}$	$-9.117e^{+02}$
ZBTB12	TTCTAGATAT	$1e^{2828}$	$-6.513e^{+03}$	SOX8	GTGAGATGAA	$1e^{-329}$	$-7.577e^{+02}$
CDX2	GAAGGCAATAAA	$1e^{-2749}$	$-6.330e^{+03}$	KLF10	ACACACCACACA	$1e^{-229}$	$-5.284e^{+02}$
ZNF740	CTCCTCACCT	$1e^{-2609}$	$-6.008e^{+03}$	ZNF263	GGTTCTCC	$1e^{-205}$	$-4.740e^{+02}$
ZFX	CTGCCTCGGCCT	$1e^{-2531}$	$-5.829e^{+03}$	NRL	ATCAGCGA	$1e^{-201}$	$-4.636e^{+02}$
GATA6	GTGGCGATATCT	$1e^{-2294}$	$-5.283e^{+03}$	CUX1	CATCATCATCAT	$1e^{-185}$	$-4.272e^{+02}$
ONECUT3	TTGATTCTTC	$1e^{-1974}$	$-4.546e^{+03}$	ZNF416	TGCCCTGC	$1e^{-183}$	$-4.225e^{+02}$
ZNF519	GGGAGGCGGA	$1e^{-1939}$	$-4.465e^{+03}$	FOXH1	CGTAGATT	$1e^{-150}$	$-3.475e^{+02}$
Nr2f2	GCTGACCCCC	$1e^{-1413}$	$-3.254e^{+03}$	HOXA1	CCATCCATCC	$1e^{-71}$	$-1.652e^{+02}$
Cux1	TGATGATGGTGG	$1e^{-775}$	$-1.785e^{+03}$	GM397	ACACACACACAC	$1e^{-32}$	$-7.548e^{+01}$

Figure S4. Transcription factor binding site motifs identified using SICER.

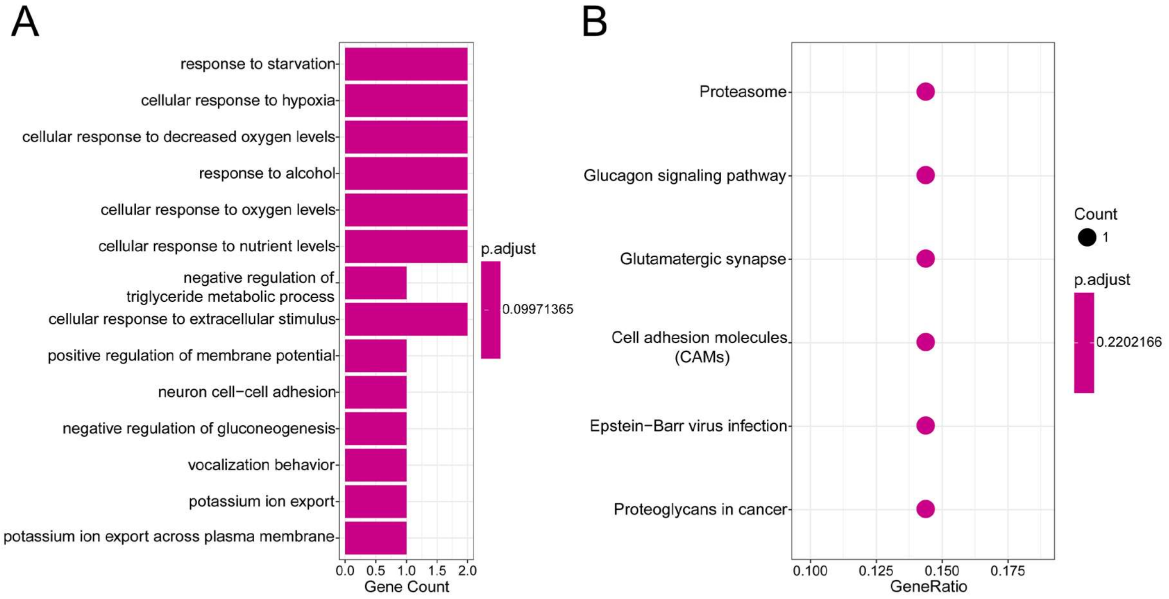

Figure S5. GO and pathway analysis by ClusterProfier package. **A** GO analysis (p.adjust<0.1). **B** Pathway enrichment (p.adjust<0.25).
